# For Personality Disorders

**DOI:** 10.1192/j.eurpsy.2022.55

**Published:** 2022-09-01

**Authors:** J. Treasure

**Affiliations:** King’s College London, Department Of Psychological Medicine, London, United Kingdom

## Abstract

The number of diagnostic categories of eating disorders have increased over time. The term transdiagnostic has been commonly used for eating disorders as in general they share problematic eating patterns and variations in weight. However, there are also extreme differences, for instance in the realm of personality style. One example of contrasts is the polygenic correlation with ADHD which is positive in binge eating disorders and absent in anorexia nervosa. This is concordant with the clinical presentation whereby AN is associated with compulsive, rigid perfectionistic features consistent with an obsessive-compulsive personality style whereas BED is associated with impulsivity. ARFID and AN have features that overlap with characteristics of autistic spectrum disorders. Nevertheless, traits of neuroticism are shared across eating disorders and other psychiatric disorders. Another contrast is in the exposure to adversity in childhood. People with binge eating disorder have many forms of childhood adversity including the ramifications of weight stigma and this leaves an imprint on personality development. Thus, there is no one size that fits all in terms of the unfolding links between personality and eating disorders.

**Disclosure:**

No significant relationships.

